# Bladder Rupture as a Complication of Circumcision following Total Subcoronal Urethral Ligation

**DOI:** 10.1155/2018/5394928

**Published:** 2018-05-31

**Authors:** Asal Hojjat, Sorena Keihani, Amir Hassan Mahboubi, Abdol-Mohammad Kajbafzadeh

**Affiliations:** Pediatric Urology Research Center, Children's Hospital Medical Center, Pediatrics Center of Excellence, Tehran University of Medical Sciences, Tehran, Iran

## Abstract

Circumcision is one of the most commonly performed procedures and generally is considered a safe and minor surgery. However, serious and catastrophic complications may sometime occur if adequate attention is not paid to all aspects of this procedure. Bleeding is the most common early complication of circumcision and sometimes is controlled by applying sutures. We hereby report a case of urethral obstruction after deep suturing of the bleeding site performed at a district center that led to bladder rupture as an exceedingly rare complication following circumcision.

## 1. Introduction

Circumcision is a common surgical procedure worldwide. Although it is generally considered a safe surgical procedure, serious complications like excessive bleeding, necrosis, and amputation of glans are reported [1]. Bladder rupture is a devastating surgical emergency in children that mostly occurs in the setting of trauma. Nontraumatic bladder rupture, on the other hand, is an extremely rare clinical entity that usually happens in chronically unused bladders or with underlying bladder disease. We hereby report bladder rupture as a rare complication of circumcision that happened following extensive suturing as an attempt to control severe postoperative bleeding.

## 2. Case Report

A 2-year-old healthy boy underwent an office-based surgical circumcision by his physician. During the procedure, profound bleeding was observed that was not controlled by applying direct pressure. The physician attempted to control the bleeding by multiple deep suturing; the bleeding was stopped successfully and the patient was discharged home. During the postoperative period, the child had progressive painful and difficult voiding with only few drops of urine after straining. During this period the child was prescribed painkillers for his discomfort and no additional evaluation was done. After about a week he was referred to a district hospital with fever (temperature=38.1C), agitation, vomiting, urinary retention, and significant abdominal distension. Abdominal examination showed decreased bowel sounds, dull percussion, and severe guarding. Urgent abdominopelvic ultrasound revealed large volume of free fluid in the abdomen and pelvis, with small amount of urine in the bladder. The initial lab tests also showed leukocytosis with left shift and increased blood creatinine and blood urea nitrogen. Catheterization with a 6-Fr feeding tube failed because it did not pass beyond the subcoronal urethra. The patient underwent emergency midline laparotomy with the diagnosis of acute abdomen and the fluid was drained. A small intraperitoneal bladder rupture was noticed at the dome of bladder. The gastrointestinal tract was inspected precisely and was intact. The diagnosis of intraperitoneal bladder rupture with urinary ascites was made probably due to near-total urethral obstruction. The rupture site was repaired in two layers and a suprapubic cystostomy catheter was fixed.

**Figure 1:**
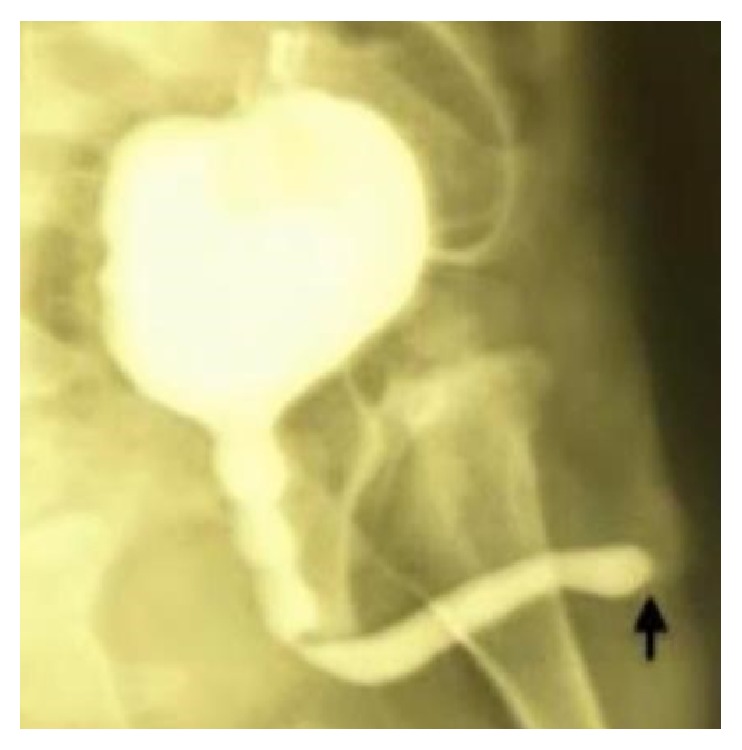
Antegrade VCUG via the suprapubic catheter after surgery for bladder rupture; full-length urethral dilation is evident up to point of obstruction (black arrow).

Two months later, the patient was referred to our center for further evaluation and treatment. Antegrade voiding cystourethrography (VCUG) was performed via the suprapubic catheter that showed terminal urethral obstruction (Figure 1). Urethroscopy was attempted under general anesthesia that failed due to complete obstruction at 1 cm from the meatus.

**Figure 2:**
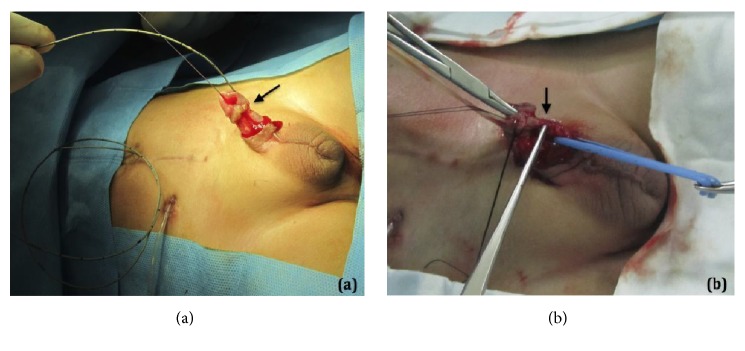
(a) A 3-Fr ureteric catheter is blocked at the obstruction point at 1 cm from urethral meatus (arrow); also note the midline scar of previous surgery and suprapubic catheter; (b) the black arrow point to the fibrotic site obstructing the urethra.

**Figure 3:**
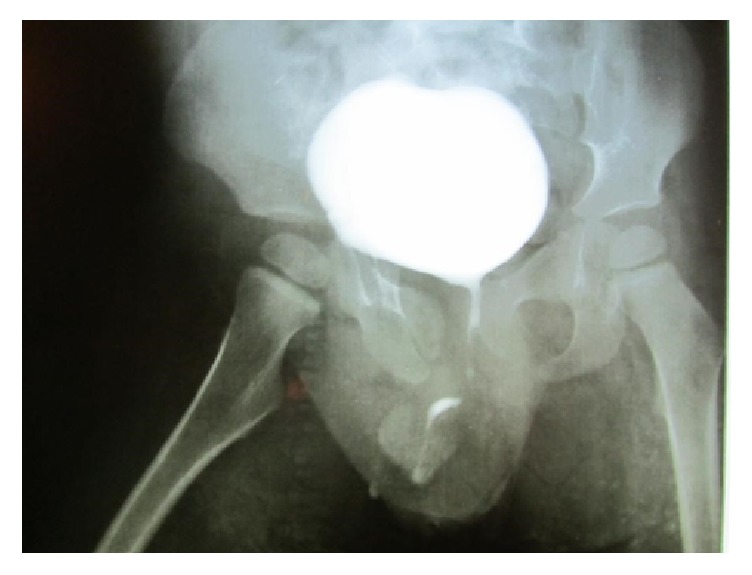
VCUG six weeks after urethral repair surgery showing normal bladder and urethra.

Decision was made to explore the area and to repair the urethra. Through a circumferential incision, distal urethra was elevated from the corpus spongiosum. A 3-Fr ureteric catheter also did not pass the obstructed part (Figure 2(a)). A 5-mm fibrotic tissue was encountered at the site of obstruction (Figure 2(b)). The corpus spongiosum was dissected free from the corpora cavernosa to prevent iatrogenic chordee after end-to-end urethral anastomosis. The obstructed fibrotic part of urethra was completely resected and an end-to-end urethral anastomosis was performed along with spongioplasty over an 8-Fr silicon catheter in two layers; dartos pedicled flap was used to cover the site of anastomosis. The postoperative period was uneventful and the patient was discharged home with suprapubic and urethral catheter. The urethral catheter was removed seven days following the surgery. The suprapubic catheter was removed four weeks after the surgery following normal urethral voiding and normal ultrasound. A VCUG was performed six weeks after the surgery that showed normal bladder and urethra with acceptable voiding per urethra (Figure 3). During a 2.5-year follow-up period, the patient was asymptomatic with normal renal function, ultrasound, and voiding pattern. He had a maximum flow rate of 15.3 ml/sec in uroflowmetry study.

## 3. Discussion

Bleeding—the most common complication of circumcision—happens in 0.08% to 2.1% of children undergoing this procedure by trained medical personnel [2, 3]. It mainly occurs as oozing or bleeding along the suture lines or from a discrete vessel, most commonly the frenular artery [1]. The majority of postcircumcision bleeding is minor and may be managed by applying direct pressure; however excessive bleeding may complicate about 0.6% of cases that may necessitate placing a suture [1, 3]. However, meticulous technique may be used and deep sutures should be avoided to prevent urethral injury or other complications like fistula formation. In the reported case, the physician used a deep suture to control the bleeding that led to near-total urethral obstruction, straining, and rise in bladder pressure that finally led to rupture at the dome of the bladder as the weakest point. Urethral obstruction rarely happens after circumcision; some serious cases of urinary retention have been reported after using PlastiBell device and in one case meatal obstruction led to septic shock, metabolic disturbances, and finally death in a neonate [4]. However, bladder rupture as a complication of circumcision is exceedingly rare. To our best knowledge only one such case is reported by Jee and Millar in a 5-year-old boy after circumcision with PlastiBell [5]. We hereby reported bladder rupture in a 2-year-old boy after circumcision that was attributable to extensive suturing of the bleeding site and obstructing the urethra. This complication could be avoided if more superficial sutures and careful technique was used to control the bleeding and if the urethral patency was confirmed after the procedure or when the child had difficult voiding during the postoperative period. This report is unique since the circumcision method was different from the previous report (surgical versus PlastiBell) and the obstruction followed suturing to control the bleeding that per se is a common complication.

Taken together some serious and even fatal complications, including urethral obstruction and bladder rupture, might occur after circumcision. Surgeons must be alert about subtle signs and symptoms that hint at complications from the very first moment following the operation. Trained medical personnel that are familiar with complications and their management should perform circumcision in medical centers and under sterile conditions. Careful techniques and postoperative supervision should be implemented to minimize the rare, though serious, complications and act in a timely manner when they occur. We therefore recommend a standardized approach in teaching all primary care pediatricians, obstetric specialists, pediatric surgeons, and all physicians and health providers routinely perform circumcision. Not only having the skills is necessary to perform this common procedure, but also a careful and comprehensive physical examination is required to manage any kind of early and late complications of circumcision. Bleeding is the most common complication in postcircumcised patients that usually can be managed by direct pressure (such as compressive gauze pads). But in more serious complications with delayed bleeding and/or unstable hemostasis, you may need to transfer patient to operating room and patient's bleeding can be managed following suture placement and/or recircumcision. In the present case, using precise small gauge dissolving suture and confirming urethral patency could have been used as warranted solutions to prevent further complications. Although circumcision is a frequent minor surgery, considering a few serious complications reported, prompt urologic consultation or referring to an upper level of care is strongly suggested in more complicated patients.
